# A radiomics-based decision support tool improves lung cancer diagnosis in combination with the Herder score in large lung nodules

**DOI:** 10.1016/j.ebiom.2022.104344

**Published:** 2022-11-10

**Authors:** Benjamin Hunter, Mitchell Chen, Prashanthi Ratnakumar, Esubalew Alemu, Andrew Logan, Kristofer Linton-Reid, Daniel Tong, Nishanthi Senthivel, Amyn Bhamani, Susannah Bloch, Samuel V. Kemp, Laura Boddy, Sejal Jain, Shafick Gareeboo, Bhavin Rawal, Simon Doran, Neal Navani, Arjun Nair, Catey Bunce, Stan Kaye, Matthew Blackledge, Eric O. Aboagye, Anand Devaraj, Richard W. Lee

**Affiliations:** a Department of Surgery and Cancer, Imperial College London, Du Cane Road, London, W12 0NN, UK; b Lung Unit, The Royal Marsden NHS Foundation Trust, Fulham Road, London, SW3 6JJ, UK; c Department of Respiratory Medicine, Charing Cross Hospital, Imperial College Healthcare Trust, Fulham Palace Road, London, W6 8RF, UK; d Department of Respiratory Medicine, University College London Hospitals NHS Foundation Trust, Euston Road, London, NW1 2BU, UK; e Department of Respiratory Medicine, Nottingham University Hospitals NHS Foundation Trust, Hucknall Road, Nottingham, NG5 1PB, UK; f Early Diagnosis and Detection Centre, The Royal Marsden NHS Foundation Trust, Fulham Road, London, SW3 6JJ, UK; g Department of Respiratory Medicine, Queen Elizabeth Hospital, Stadium Road, Woolwich, London, SE18 4QH, UK; h Department of Radiology, The Royal Brompton and Harefield Hospitals, Guy's and St Thomas's NHS Foundation Trust, Sydney Street, London, SW3 6NP, UK; i CRUK Cancer Imaging Centre, The Institute of Cancer Research, Cotswold Road, Sutton, SM2 5NG, UK; j Department of Radiology, University College London Hospitals NHS Foundation Trust, Euston Road, London, NW1 2BU, UK; k Clinical Trials Unit, The Royal Marsden NHS Foundation Trust, Downs Road, Sutton, SM2 5PT, UK; l Department of Medical Oncology, The Royal Marsden NHS Foundation Trust, Downs Road, Sutton, SM2 5PT, UK; m Computational Imaging Group, The Institute of Cancer Research, Cotswold Road, Sutton, SM2 5NG, UK; n National Heart and Lung Institute, Imperial College London, Guy Scadding Building, Dovehouse Street, London, SW3 6LY, UK

**Keywords:** Lung cancer, Radiomics, Early diagnosis, Machine learning, Lung nodules, Deep learning

## Abstract

**Background:**

Large lung nodules (≥15 mm) have the highest risk of malignancy, and may exhibit important differences in phenotypic or clinical characteristics to their smaller counterparts. Existing risk models do not stratify large nodules well. We aimed to develop and validate an integrated segmentation and classification pipeline, incorporating deep-learning and traditional radiomics, to classify large lung nodules according to cancer risk.

**Methods:**

502 patients from five U.K. centres were recruited to the large-nodule arm of the retrospective LIBRA study between July 2020 and April 2022. 838 CT scans were used for model development, split into training and test sets (70% and 30% respectively). An nnUNet model was trained to automate lung nodule segmentation. A radiomics signature was developed to classify nodules according to malignancy risk. Performance of the radiomics model, termed the large-nodule radiomics predictive vector (LN-RPV), was compared to three radiologists and the Brock and Herder scores.

**Findings:**

499 patients had technically evaluable scans (mean age 69 ± 11, 257 men, 242 women). In the test set of 252 scans, the nnUNet achieved a DICE score of 0.86, and the LN-RPV achieved an AUC of 0.83 (95% CI 0.77–0.88) for malignancy classification. Performance was higher than the median radiologist (AUC 0.75 [95% CI 0.70–0.81], DeLong p = 0.03). LN-RPV was robust to auto-segmentation (ICC 0.94). For baseline solid nodules in the test set (117 patients), LN-RPV had an AUC of 0.87 (95% CI 0.80–0.93) compared to 0.67 (95% CI 0.55–0.76, DeLong p = 0.002) for the Brock score and 0.83 (95% CI 0.75–0.90, DeLong p = 0.4) for the Herder score. In the international external test set (n = 151), LN-RPV maintained an AUC of 0.75 (95% CI 0.63–0.85). 18 out of 22 (82%) malignant nodules in the Herder 10–70% category in the test set were identified as high risk by the decision-support tool, and may have been referred for earlier intervention.

**Interpretation:**

The model accurately segments and classifies large lung nodules, and may improve upon existing clinical models.

**Funding:**

This project represents independent research funded by: 1) Royal Marsden Partners Cancer Alliance, 2) the 10.13039/100016916Royal Marsden Cancer Charity, 3) the 10.13039/501100000272National Institute for Health Research (NIHR) Biomedical Research Centre at the Royal Marsden NHS Foundation Trust and The Institute of Cancer Research, London, 4) the 10.13039/501100000272National Institute for Health Research (NIHR) Biomedical Research Centre at 10.13039/501100000761Imperial College London, 5) 10.13039/501100000289Cancer Research UK (C309/A31316).

**Research in context**

**Evidence before this study**

The current guidelines for investigating lung nodules rely on clinical risk models, such as the Brock and Herder scores, and most nodules above 15 mm will trigger the 10% threshold for investigation. Many large nodules fall into the 10–70% Herder category, wherein the British Thoracic Society Guidelines suggest a broad range of options, from surveillance to surgery, and methods to improve stratification are needed. In the many years since the Herder model was developed, few studies have investigated how it could integrate with non-invasive radiomics models to improve early cancer diagnosis rates, and no existing studies have looked at large (15–30 mm) nodules only.

**Added value of this study**

This study developed a radiomics-based cancer prediction model in 15–30 mm lung nodules, which are not stratified well by existing guidelines. The developed model, termed the large-nodule radiomics predictive vector, achieved higher cancer prediction accuracy than the Brock score, and by integrating with the Herder model, would have led to early intervention in 82% of the malignant nodules with Herder scores of 10–70%. Because the model requires fewer variables than the Brock and Herder scores, it could potentially streamline the risk-classification process for clinicians in the future, particularly where PET scanning is not available or will be delayed. The use of a highly-accurate deep learning segmentation pipeline means that the model is not dependent on human nodule segmentation.

**Implications of all the available evidence**

The large nodule radiomics model improves upon or extends existing clinical models, and integrates with the British Thoracic Society guidelines to provide net-benefit in terms of early cancer intervention. Although prospective evaluation is needed, this tool may aid clinician decision making with regards to large lung nodules in the future.

## Introduction

Incidental lung nodules are a common finding on CT scans. Most are benign, but some represent early-stage cancers and provide an opportunity for early lung cancer diagnosis.^1^ Correctly stratifying nodules is challenging, because triaging a high-risk nodule as low-risk could lead to delayed cancer diagnosis, but over-investigating low-risk nodules may expose patients to undue complications. Therefore many guidelines have been developed to support management decisions, which incorporate nodule size as a key risk-factor.^2^, ^3^, ^4^, ^5^, ^6^, ^7^ The American College of Radiology Lung-RADS screening criteria place solid nodules ≥15 mm into the highest risk category (4B), recommending consideration of biopsy.^7^ A 15 mm threshold is supported by data from a study of 2821 nodules, which found that clinical risk factors for malignancy differed above and below this cut-off in multivariable regression.^8^ Nevertheless, the malignancy rate in ≥15 mm/Lung-RADS 4B nodules is still variable (23.5% - 36.3%), and additional non-invasive biomarkers may help to identify those most at risk.^9^^,^^10^

In the United Kingdom (U.K.), the British Thoracic Society (BTS) guidelines are used to investigate incidental nodules.^3^ These guidelines use a Brock score threshold of ≥10% to trigger further investigation of solid nodules, which would be met by a 50-year-old woman with a 15 mm nodule and no other risk factors, and may therefore not stratify large (15–30 mm) nodules well.^11^ The BTS algorithm also incorporates the Herder score, which utilises PET-data.^12^ The original Herder model was developed 17 years ago in a small patient cohort, but remains a central component of nodule multidisciplinary meetings across the U.K.^3^ Patients within the 10–70% Herder category have a broad range of possible clinical actions, and methods to better stratify this group are needed. Although Herder has been validated in modern datasets, it remains to be seen how machine-learning based approaches could enhance the model to improve patient stratification.^13^

The requirement for additional decision-support is particularly important following the COVID-19 pandemic, which caused disruption to diagnostic services.^14^ This may be especially relevant in the U.K., where PET availability lags behind other European countries, and may not be routinely available at all centres.^15^ Finally, because Brock and Herder both require a large number of clinical variables, they can be time consuming to calculate, and non-invasive methods with fewer data points could streamline the decision-making process for clinicians.

Many radiomics models for nodule classification have been developed in recent years.^16^, ^17^, ^18^, ^19^, ^20^, ^21^, ^22^ Baldwin et al. validated a lung nodule convolutional neural network (LN-CNN) in 1187 patients with 5–15 mm nodules, achieving an AUC of 89.6%.^21^ A broad range of different size criteria have been utilised, including mixed (5–30 mm) and small nodule only (5–15 mm) cohorts, but no studies have explored the utility of radiomics in large (≥15 mm) nodules, where malignancy risk is highest but still variable.^9^ Given that the aetiology and risk may differ, and that models perform best on data resembling the training cohort, we hypothesise that a 15–30 mm nodule model may be able to integrate with the Herder score to improve early diagnosis.

Through the Lung Imaging Biobank for Radiomics and AI research (LIBRA), we aimed to develop a pipeline for multi-centre radiomics research capturing real-world, heterogeneous data, and to create a radiomics algorithm to accurately classify large lung nodules according to cancer risk. Finally, we sought to develop a decision-support tool to reduce delayed cancer diagnosis rates in the broad 10–70% Herder risk group.

## Methods

### Ethics

**Fig. 1:**
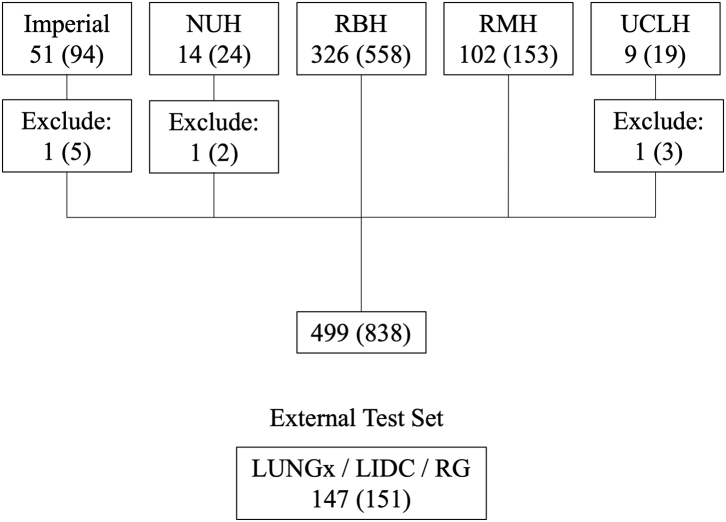
**Study recruitment diagram.** The numbers of scans are shown in parentheses. Three patients could not be analysed for technical reasons, leading to a final subset of 499 patients and 838 scans. Abbreviations: NUH, Nottingham University Hospital; RBH, The Royal Brompton Hospital; RMH, The Royal Marsden Hospital; UCLH, University College London Hospitals; LIDC, Lung-image database consortium; RG, Non-small cell lung cancer Radiogenomics study.

Health Regulatory Authority (HRA) and research ethics committee (REC) approval were obtained for the Lung Imaging Biobank for Radiomics and AI (LIBRA) retrospective cohort study (IRAS ID: 274775, REC reference 20/NI/0088, ClinicalTrials.gov: NCT04270799). Patient consent was not required. Patients were recruited between 1st July 2020 and 1st April 2022 by the clinical teams at participating centres (Fig. 1).

### Study sample

Inclusion criteria:
  • Age >18
  • Baseline CT reporting 15–30 mm pulmonary nodules.
  • Ground truth: Benign: either scan data showing stability for 2 years (based on diameter) or one year (based on volumetry), or resolving. Sub-solid nodules required stability for 4 years. Malignant: biopsy-proven.
Exclusion criteria:
  • Absence of analysable scans.
  • Slice thickness >2.5 mm.
  • Ground truth unknown.

Up to three CT scans (baseline, interim and the final follow-up) were included for each patient. The final diagnosis at the last scan defined the ground truth for all other scans for each patient. Only a single nodule per scan was included.

Patients were identified for the external test set from the LIDC-IDRI, LUNGx and NSCLC radiogenomics public data sets.^23^, ^24^, ^25^ Because of the small number of eligible patients in the LUNGx and LIDC data sets, up to two nodules per patient were included, leading to a total of 151 nodules in 147 patients. Clinicodemographic data for the external test set are shown in Supplementary Table S1.

### Data anonymisation and storage

CT scans were link-anonymised at local centres using DICOM Browser or centre-specific methods where required. Anonymised DICOM images and demographic data were uploaded to the LIBRA XNAT server.

### Radiologist benchmarking

The 252 test-set scans were reviewed by three clinical radiologists: two were post-FRCR with over 5 years of experience (MC, EA) and one was pre-FRCR with 3 years of experience (AL). The readers were blinded to clinical data including the malignancy status, but were able to see the entire CT scan including the background lung parenchyma. Scans were rated using a 5-point scale: 1 – benign, 2 – probably benign, 3 – indeterminate, 4 – probably malignant and 5 – malignant. Receiver-Operator Characteristic (ROC) curves were then constructed to calculate AUCs.

### Image pre-processing

DICOM images were converted to nifti format using dcm2niix (https://github.com/rordenlab/dcm2niix). Manual segmentation was performed by a post-FRCR clinical oncologist with 7 years’ experience (BH) using ITK-Snap.

Images and segmentations were resampled to 1 × 1 × 2 mm voxel dimensions using cubic spline and nearest neighbour interpolation respectively. Intensity values were capped at −2000 to +2000.

### Radiomics model development

Data were randomly split into training and test sets (70% and 30% respectively) using the sample.split R function, maintaining equal proportions of malignant nodules. The split was grouped by study ID to prevent data leakage when multiple scans were present for a given patient.

Radiomic features were extracted using TexLab 2.0, developed in MATLAB 2015b (Mathworks Inc., Nathick, Massachusetts, USA) using 25HU intensity bins. TexLab initially extracts 666 features, including high-order wavelet transformations. To improve study interpretability, we removed wavelet features prior to model development. The 82 remaining features were scaled using Z-standardisation (*X*− $\bar{X}$/*SD*). Univariable logistic regression was performed for each feature against the cancer status, and those with p values < 0.05 (Wald test) after Benjamini–Hochberg (B–H) correction were selected for the LASSO logistic regression model. Ten-fold cross-validation was used to select the largest value of lambda giving a cross-validated error within one standard error of the minimum (lambda.1se). The weighted sum of features with non-zero coefficients yielded the large-nodule radiomics predictive vector (LN-RPV).

ROC-curves were constructed using the cutpointr package (https://cran.r-project.org/web/packages/cutpointr/). Optimal cutpoints were selected to maximise the Youden index (sensitivity + specificity −1) for malignancy prediction.

K-means clustering was used to divide the training-set into low and high-risk subgroups based on the RPV (Supplementary Fig. S1). The same criteria were applied to the test set.

### Auto-segmentation

Scans and masks were cropped to the maximal 3D segmentation dimensions. For auto-segmentation, we used the nnUNet, a self-calibrating network that automates hyperparameter optimisation and 5-fold cross-validation.^26^ Each fold was trained for 1000 epochs before hyperparameter selection. Training and test set performance were evaluated using the DICE score.

### Clinical modelling

Variables required for Brock and Herder calculation were obtained from patient records. For the purpose of Herder calculation, patients with no recorded PET data were taken to be PET negative.

Univariable logistic regression was performed to select predictive clinical features (Wald test p < 0.001 after B–H correction). Categorical variables were converted to dummy variables prior to training, with the most common level becoming the reference standard. Multivariable logistic regression models were developed incorporating the LN-RPV and statistically significant clinical features.

For comparison of the radiomics model with the Brock and Herder scores, we used a subset of the 252 test set scans pertaining to only baseline CT images containing solid nodules (n = 117), which match the ‘initial approach to solid pulmonary nodules’ algorithm of the BTS guidelines.

**Fig. 2:**
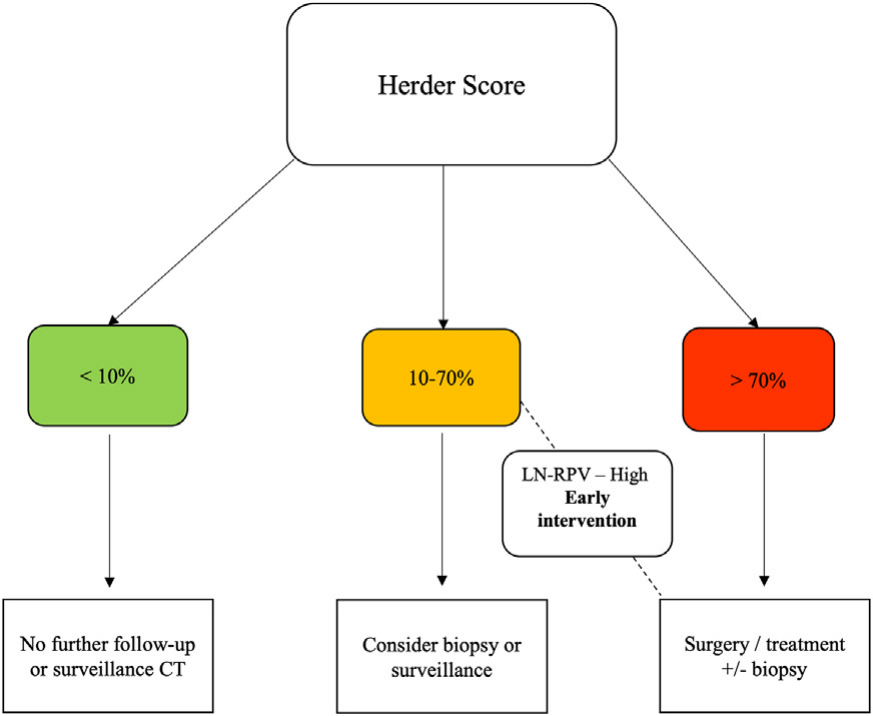
**Radiomics decision-support tool.** The large-nodule radiomics-predictive vector (LN-RPV) is used to prompt earlier intervention in patients with intermediate (10–70%) Herder scores but a high-risk radiomics score.

To assess the impact of the LN-RPV, we devised a decision-support tool to assess how the model could reduce missed diagnoses or delayed treatment associated with the 10–70% Herder score category (Fig. 2). Decision support impact was modelled for solid nodules in the test set (n = 174).

### Statistical analysis

Analyses were performed in R Studio (v1.3.1073) and Python (v3.7.3). Due to the exploratory nature of this work, the target recruitment size was based on expert consensus only. All p values were two sided, and a cut-off of 0.05 was used for statistical significance. 95% confidence intervals for AUC values were obtained via bootstrapping with 1000 iterations. DeLong's test was used to compare model ROC curves. As the distribution of the LN-RPV was non-normal, we used the Kruskal–Wallis test to look for interactions between scan vendor and LN-RPV. Intra-class correlation co-efficients (ICCs) were analysed using the icc R function with the following parameters: Model: two-way, type: agreement, and unit: single.

### Role of funders

This project represents independent research funded by: 1) Royal Marsden Partners Cancer Alliance, 2) the Royal Marsden Cancer Charity, 3) the National Institute for Health Research (NIHR) Biomedical Research Centre at the Royal Marsden NHS Foundation Trust and The Institute of Cancer Research, London, 4) the National Institute for Health Research (NIHR) Biomedical Research Centre at Imperial College, London, 5) Cancer Research U.K. (C309/A31316). The funders had no role in study design, data collection, data analysis or manuscript writing.

## Results

### Patient and scan characteristics

Overall, we recruited 502 patients, of whom 499 had evaluable scans. Of the 499, the mean age was 68.94 (± SD 10.73), with 257 male and 242 female patients. Table 1 shows the distribution of clinicodemographic features amongst the training and test sets at the scan level.

**Table 1:** Patient clinicodemographic features (presented at the scan level).

Characteristic	Training	Test	Overall
**Age** (mean, SD)	69.02 (10.47)	69.23 (9.65)	69.08 (10.23)
**Gender** (n, %)			
Male	280 (47.8)	131 (52.0)	411 (49.0)
Female	306 (52.2)	121 (48.0)	427 (51.0)
**Nodule size** (mean, SD)	20.35 (4.56)	20.87 (4.99)	20.51 (4.70)
**Nodule density** (n, %)			
Solid	418 (71.3)	174 (69.0)	592 (70.6)
Sub-solid	126 (21.5)	65 (25.8)	191 (22.8)
GGO	42 (7.2)	13 (5.2)	55 (6.6)
**Spiculation** (n, %)			
Yes	120 (20.5)	80 (31.7)	200 (23.9)
No	466 (79.5)	172 (68.3)	638 (76.1)
**Malignancy** (n, %)			
Yes	366 (62.5)	158 (62.7)	524 (62.5)
No	220 (37.5)	94 (37.3)	314 (37.5)
**PET avidity** (n, %)			
Absent	35 (6.0)	18 (7.1)	53 (6.3)
Faint	116 (19.8)	33 (13.1)	149 (17.8)
Moderate	146 (24.9)	63 (25.0)	209 (24.9)
Intense	95 (16.2)	60 (23.8)	155 (18.5)
Not recorded	194 (33.1)	78 (37.0)	272 (32.5)
**Smoking** (n, %)			
Never	94 (16.0)	22 (8.7)	116 (13.8)
Ex/Current	403 (68.8)	181 (71.8)	584 (69.7)
Unknown	89 (15.2)	49 (9.5)	138 (16.5)
**Lung disease** (n, %)			
Yes	323 (55.1)	146 (57.9)	469 (56.0)
No	263 (44.9)	106 (42.1)	369 (44.0)
**Previous lung ca** (n, %)			
Yes	25 (4.3)	7 (2.8)	32 (3.8)
No	561 (95.7)	245 (97.2)	806 (96.2)
**FH of lung ca** (n, %)			
Yes	7 (1.2)	3 (1.2)	10 (1.2)
No	579 (98.8)	249 (98.8)	828 (98.8)
**Previous non-lung ca** (n, %)			
Yes	131 (22.4)	60 (23.8)	191 (22.8)
No	455 (77.6)	192 (76.2)	647 (77.2)

Abbreviations: SD, Standard Deviation, GGO, ground-glass opacity, ca,cancer.

The overall proportion of benign vs malignant nodules was 37.5 vs 62.5% respectively. The data set included a mixture of solid (70.6%), subsolid (22.8%) and ground-glass opacities (6.6%), with proportions well balanced amongst the training and test sets. CT scans were acquired from five institutions and four scan vendors (GE Medical System, Philips, Siemens and Toshiba). 464 (55%) scans were non-contrast, and a large mixture of soft and sharp reconstruction kernels were included (Table 2).

**Table 2:** Scan vendor and reconstruction kernels (n = 838).

Kernel	Frequency
**GE medical systems**	
STANDARD	52
LUNG	30
CHST	24
SOFT	10
BONEPLUS	17
ULTRA	1
DETAIL	1
**Philips**	
B	38
C	4
E	1
L	19
YA-C	21
**Siemens**	
l30-70/2-3	79
B20-50f/s	160
B60-65f/s	38
B70-80f	271
D30f	1
**Toshiba**	
FC03-13	31
FC30-52	29
FC83-86	11

### Radiologist Performance Benchmarking

**Fig. 3:**
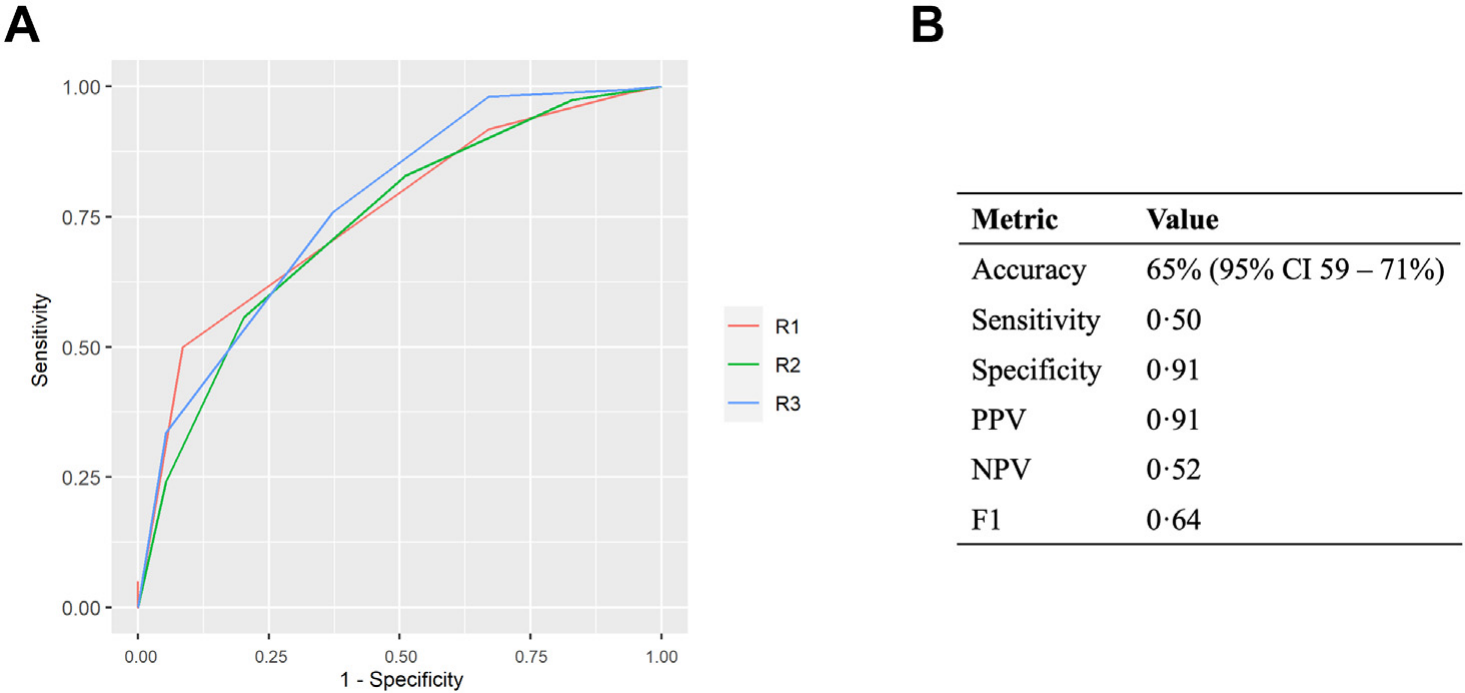
**Radiologist Performance Benchmarking (n = 252).** a) Malignancy-prediction ROC curves for the three radiologists in the test set. The AUCs were: R1: 0.75 (95% CI 0.70–0.81), R2: 0.74 (95% CI 0.67–0.79) and R3: 0.77 (95% CI 0.71–0.82). b) Malignancy prediction performance metrics for the radiologist with the median AUC (R1) after selecting the optimum cut-point to maximise the Youden index (4 – Probably malignant). The radiologist achieved an accuracy of 65% (95% CI 59–71%). Abbreviations: AUC, Area under the curve; PPV, Positive predictive value; NPV, Negative predictive value; CI, Confidence interval.

In the test set, the three readers achieved AUCs of 0.75 (95% CI 0.70–0.81), 0.74 (95% CI 0.67–0.79) and 0.77 (95% CI 0.71–0.82) for lung nodule malignancy classification (Fig. 3a). The performance metrics for the radiologist with the median AUC (R1) were: accuracy 65% (95% CI 59–71%), sensitivity 0.50, specificity 0.91, PPV 0.91, NPV 0.52 and F1 0.64. Metrics for all three radiologists are provided in Supplementary Table S2.

### LN-RPV performance

**Fig. 4:**
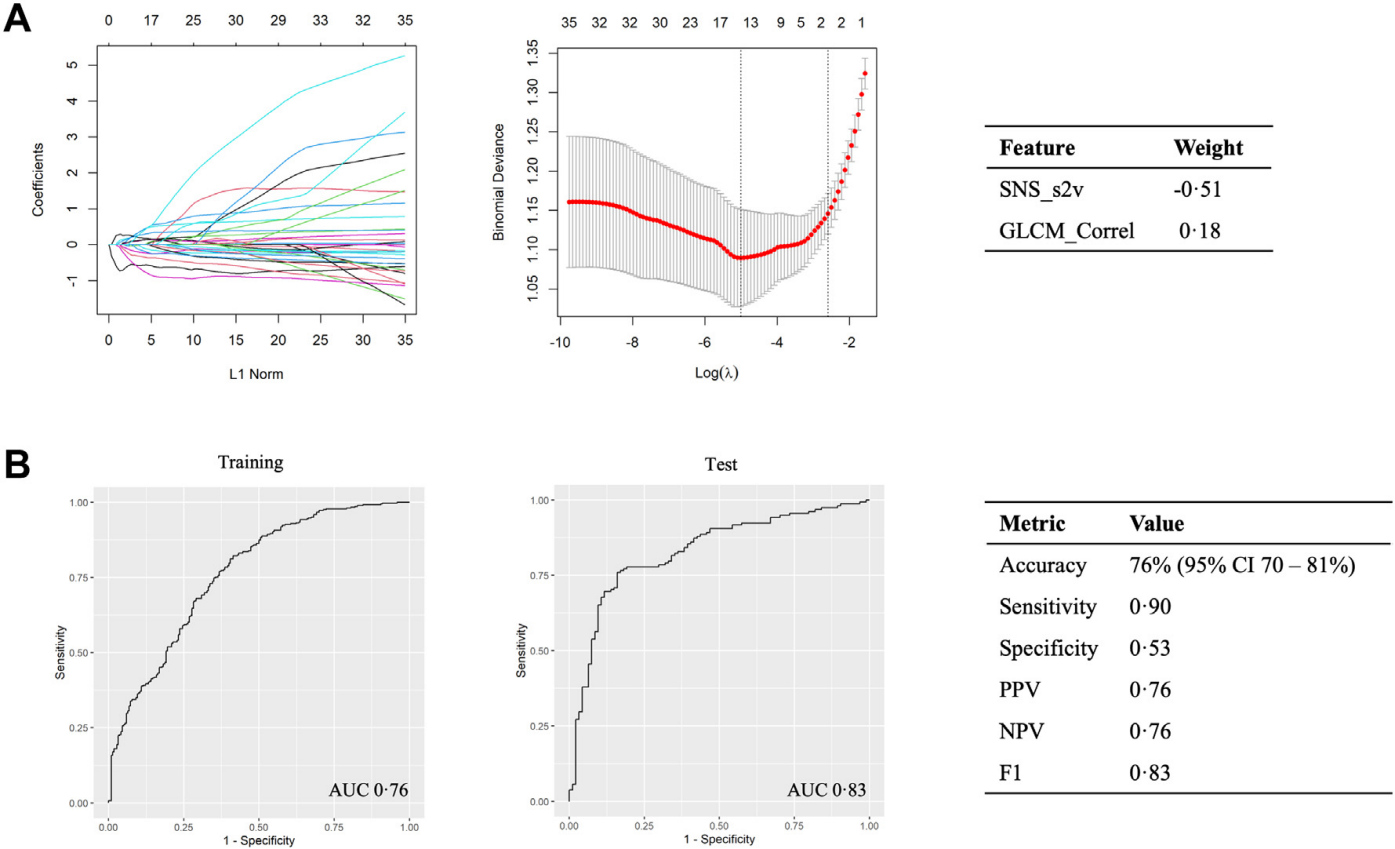
**The Large-Nodule Radiomics Predictive Vector (LN-RPV).** a) The LASSO regularisation plot, lambda plot and the regression weights for the two selected features are shown. b) ROC-AUC curves for malignancy prediction were used to select cut-offs based on the training-set Youden index. In the test set, the model achieved an AUC of 0.83 (95% CI 0.77–0.88) and an accuracy of 76% (95% CI 70–81%). Abbreviations: AUC, Area under the curve; PPV, Positive predictive value; NPV, Negative predictive value, CI, Confidence interval.

The cross-validated LASSO model retained two features with non-zero coefficients (Fig. 4a). The regression formula to generate the LN-RPV was thus: SNS_s2v∗-0.5143257 + GLCM_Correl∗ 0.1840902.

The LN-RPV AUC was 0.76 (95% CI 0.73–0.80) in the training set and 0.83 (95% CI 0.77–0.88) in the test set. The threshold which yielded the maximum Youden index in the training set was −0.1991184. Using this threshold, the model achieved an accuracy of 76% (95% CI 0.70–0.81), sensitivity and specificities of 90% and 53% respectively, and an F1 score of 0.83 in the test set (Fig. 4b).

We tested the LN-RPV against axial diameter or volume alone. For diameter, training and test set AUCs were 0.58 (95% CI 0.53–0.63) and 0.56 (95% CI 0.49–0.63) respectively. For volume, training and test set AUCs were 0.70 (95% CI 0.66–0.75) and 0.76 (95% CI 0.70–0.82) respectively. LN-RPV performance was statistically significantly higher than the median radiologist (p = 0.03), diameter (p < 0.001) and 3D volume (p = 0.001) using DeLong's test.

### Clinical modelling

A total of 14 clinical variables were assessed by univariable regression against cancer status (Table 3). 6 of these features were highly significant (p < 0.001, Wald test) after correction for multiple testing, and were selected for the multivariable model: Brock score, Herder score, a history of lung disease, a history of extra-thoracic malignancy, nodule density and PET avidity. Because of the potential issue of collinearity between the Brock, Herder and PET status, we calculated the Variance Inflation Factor (VIF) for each feature. The VIF value was 2.12 for the Brock score. The values for the Herder score, moderate and intense PET avidity were 31, 25 and 19, suggesting a high level of collinearity between Herder and PET status. Therefore, PET status was removed from the model.

**Table 3:** Univariable logistic regression results for clinical features against cancer status in the training set (n = 586, arranged by adjusted P value).

Variable	Beta	P	OR (95% CI)
Brock	0.03	<0.001	1.03 (1.02–1.04)
Herder	0.03	<0.001	1.03 (1.02–1.03)
Lung disease	−0.68	<0.001	0.51 (0.36–0.71)
Extra-thoracic cancer	1.10	<0.001	3.00 (1.88–4.79)
PET avidity	1.46	<0.001	4.30 (3.01–6.14)
Subsolid or GGO density	1.45	<0.001	4.25 (2.7–6.66)
Age	0.02	0.02	1.02 (1.00–1.04)
Nodule count	−0.46	0.02	0.63 (0.45–0.89)
Smoking status	0.38	0.02	1.46 (1.08–1.98)
Spiculation	0.57	0.02	1.77 (1.14–2.75)
Previous lung cancer	0.91	0.09	2.49 (0.92–6.72)
Gender	0.24	0.18	1.27 (0.91–1.77)
Upper lobe	0.11	0.55	1.12 (0.80–1.57)
FH of lung cancer	0.04	0.94	1.04 (0.36–3.00)

6 features had p values < 0.001 (Wald test) and were selected for the multivariable model. Abbreviations: OR: Odds ratio; CI: confidence interval; GGO: ground-glass opacity; FH: Family history.

The results of the multivariable analysis including clinical features and the LN-RPV are shown in Table 4. The Brock score was non-significant (p 0.63, Wald test). The highest feature weights were LN-RPV (0.25), sub-solid density (0.23) and ground glass density (0.21). Both LN-RPV and the Herder score had p values < 0.001, but the Herder had a low weight of 0.004. In the test set, the combined clinical-radiomics model did not perform better than the LN-RPV model alone (AUC 0.82, 95% CI 0.76–0.87, DeLong p = 0.56). We also developed fusion models incorporating both the Herder score and radiomics features (Supplementary Figs. S3 and S4). Early and late fusion models were not statistically significantly better than the Herder score alone.

**Table 4:** Multivariable logistic regression model incorporating LN-RPV and clinical features in the training set (n = 586, arranged by p value).

Variable	Beta	OR (95% CI)	P value
LN-RPV	0.25	1.28 (1.21–1.35)	<0.001
Herder	0.004	1.00 (1.00–1.01)	<0.001
Density – subsolid	0.23	1.26 (1.16–1.37)	<0.001
Density – GGO	0.21	1.24 (1.09–1.40)	<0.001
Extra-thoracic cancer	0.08	1.09 (1.01–1.18)	0.03
Absent lung disease	0.06	1.06 (1.00–1.13)	0.09
Brock	−0.001	1.00 (1.00–1.00)	0.63

The Brock score and absent lung disease lost significance in multivariable testing. LN-RPV, subsolid and GGO density had the highest beta coefficients. Abbreviations: GGO, Ground-glass opacity; OR, Odds ratio; CI, Confidence interval.

**Fig. 5:**
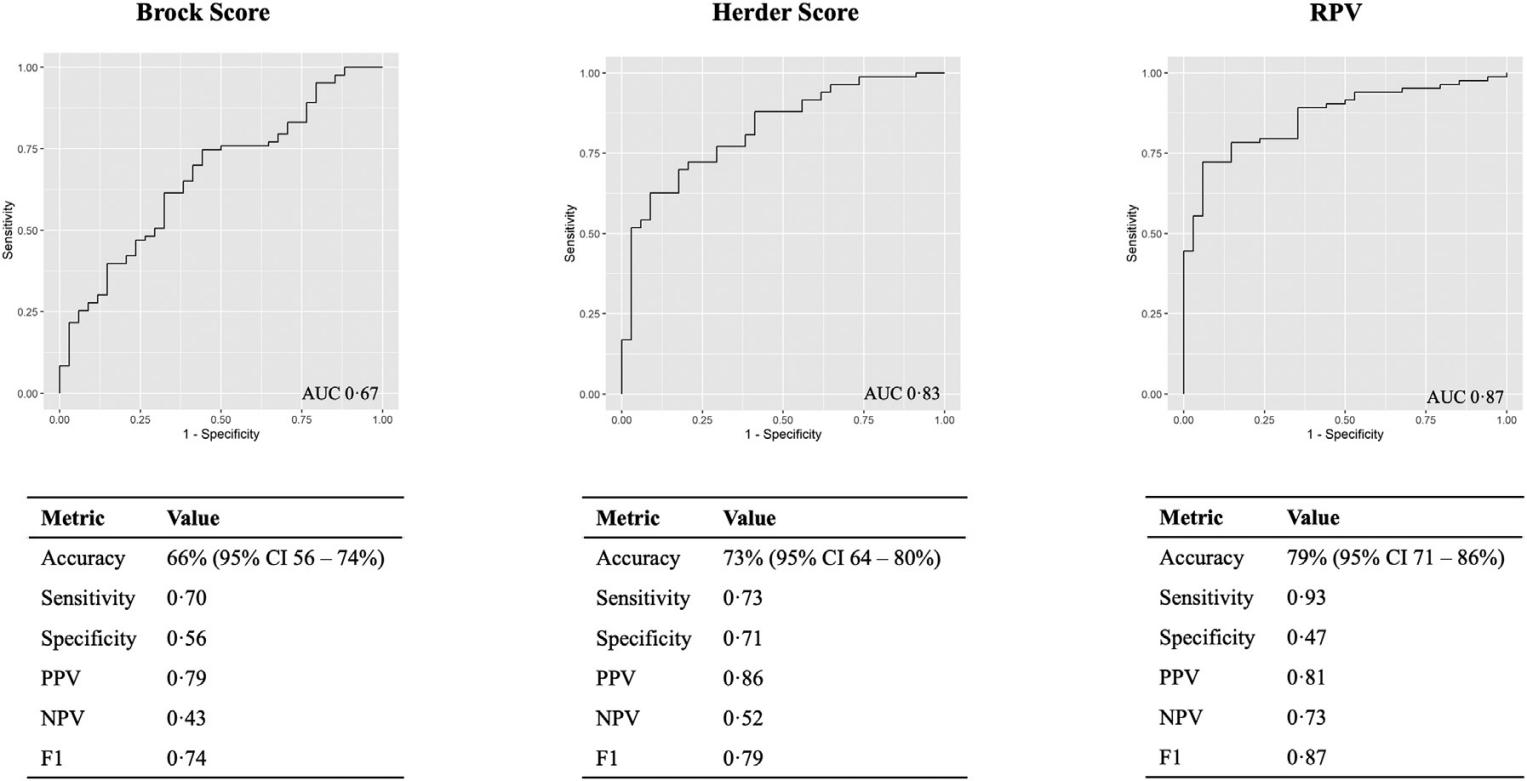
**Performance comparison between the LN-RPV and the Brock and Herder Scores for baseline solid nodules in the test set (117 patients).** Performance metrics are reported using training set cut-offs to maximise the Youden index. For accuracy, 95% CIs are given in parentheses. Abbreviations: AUC, Area under the curve; PPV, Positive predictive value; NPV, Negative predictive value; CI, Confidence interval.

The performance of the LN-RPV was compared against two commonly used clinical risk scores, Brock and Herder, for baseline solid nodules in the test set (n = 117, Fig. 5). In this cohort, the LN-RPV AUC was 0.87 (95% CI 0.80–0.93) compared to 0.67 (95% CI 0.55–0.76, DeLong p = 0.002) for the Brock score and 0.83 (95% CI 0.75–0.90, DeLong p = 0.40) for the Herder score.

### Auto-segmentation performance

**Fig. 6:**
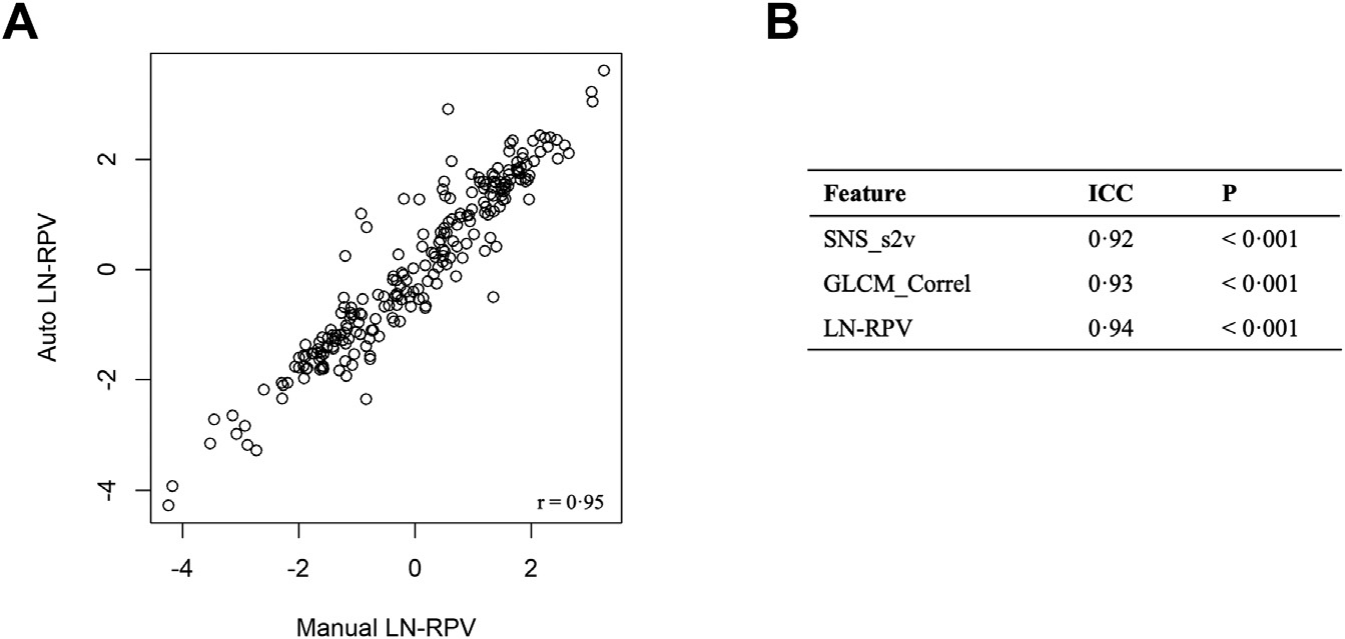
**Reproducibility of the LN-RPV using auto-segmentation.** a) Scatterplot showing high correlation between the manual and automated LN-RPV values (r = 0.95). b) Intra-class correlation co-efficients for each radiomics feature comprising the LN-RPV, and the LN-RPV alone. P values are reported for the null hypothesis that there is no-correlation between the two methods. Abbreviation: ICC, Intra-class correlation coefficient.

Example test set nnUNet nodule segmentation masks are shown in Supplementary Fig. S2. The model achieved a DICE score of 0.86 (SE 0.005). To evaluate the effect of the nnUNet auto-segmentation on the LN-RPV, features were extracted using manual and automated segmentation methods for comparison in the test set (Fig. 6). There was high correlation between the manual and automated LN-RPV (r = 0.95), with an ICC of 0.94, suggesting very high concordance between the segmentation methods.

### Radiomics feature robustness

There was no statistically significant interaction between scan vendor and LN-RPV (p = 0.46, Kruskal–Wallis test).

### Clinical decision-support

There were 38 solid test set nodules with a herder score <10%. The cross-tabulation of LN-RPV and Herder risk groups is shown for solid nodules in the test set with Herder scores ≥10% (n = 136) in Table 5. Of the 39 nodules with a Herder score of 10–70%, there were 22 malignant nodules (56%). 18 (82%) of these malignant nodules had a high LN-RPV, and would have been upgraded to early intervention using the decision-support tool.

**Table 5:** Comparison of LN-RPV and Herder categories for solid nodules in the test set with Herder scores ≥10% (n = 136).

Herder Score	LN-RPV	
	Low	High
10–70%	19 (15/4)	20 (2/18)
>70%	16 (11/5)	81 (12/69)

LN-RPV risk groups were designated as low or high based on K-Means clustering groups. The number of actual benign and malignant nodules are presented as *(benign/malignant)* for each combination of Herder and model risk groups.

### External validation

In the external test set, the LN-RPV achieved an AUC of 0.75 (95% CI 0.63–0.85) with an accuracy of 77% (95% CI 70–84%), sensitivity 84%, specificity 39%, PPV 89%, NPV 31% and F1 of 0.86. The nnUNet auto-segmentation model retained high-performance in external data (DICE 0.87).

## Discussion

Through the LIBRA study we have established a non-commercial, national pipeline for AI-based lung cancer early diagnosis research, incorporating heterogenous data from multiple institutions and scan vendors. Using this data, we developed the LN-RPV, an artificial-intelligence algorithm targeted specifically at large lung nodules, where many patients fall into a middle Herder category of 10–70% with variable management options. The LN-RPV performed better than the median radiologist and can be integrated with the BTS guidelines to reduce the risk of delayed cancer treatment.

Previous studies have reported that the Brock score has good predictive utility outside of the screening setting, which was not replicated in our incidental large nodule cohort.^27^ For solid nodules in the test set, we found that the Brock score was only moderately discriminative (AUC 0.67). This may support the hypothesis that it does not perform as well for large nodules, though the original model was intended for screening populations. The Herder score had better performance (AUC 0.83), but did not outperform the LN-RPV (AUC 0.87), which would have led to earlier intervention in 82% of the malignant nodules with Herder scores of 10–70%. As the BTS algorithm and Herder score are used widely across the UK for nodule stratification, our model has the potential to improve early cancer diagnosis and treatment by highlighting which patients are high-risk and recommending they be fast-tracked to intervention. As the LN-RPV consists of only two features, compared to the 7 values input for Herder, it could potentially streamline or automate the process of nodule risk calculation (albeit with the caveat that it requires an image-analysis pipeline). Moreover, for centres without routine access to PET, or where PET scanning will be delayed, the LN-RPV could give an earlier indication of malignancy probability. We also note the wide variability in Herder score AUCs in the literature, which likely reflects the qualitative nature of PET reporting, and may suggest Herder performance is less reliable outside of expert centres.^13^^,^^28^ Through incorporation with the nnUNet model, we have minimised the model's dependency on manual segmentation, which may allow easier integration into clinical workstreams in the future.

The first feature comprising the LN-RPV is the nodule surface-to-volume ratio, defined as the surface area divided by the total volume (SNS_s2v). The second feature is the gray-level co-occurrence matrix (GLCM) correlation (GLCM_Correl). GLCMs describe counts of co-occurring voxel gray-level intensities at given angles within the image, and the correlation metric assesses the linear dependency of gray-level values to their voxels within the GLCM. GLCM features have been used to classify benign and malignant lesions in other disease groups, including breast cancer.^29^ In non-small cell lung cancer (NSCLC), GLCM features are associated with the degree of tumour immune-infiltration, PDL1 expression and patient survival.^30^ Taken together, we hypothesise that the LN-RPV reflects the degree of nodule diffuseness and intra-tumoural heterogeneity, and could relate to spatial differences in tumour hypoxia or immune infiltration.^30^^,^^31^

In recent years, many lung nodule radiomics studies have been published, spanning a range of nodule sizes.^32^, ^33^, ^34^, ^35^, ^36^ Liu et al. developed a pre-operative radiomics nomogram using 875 patients with ≤30 mm nodules from a single centre, with a validation AUC of 0.81.^37^ The final feature set consisted of 20 features, four of which were shape or first order related, with the remainder consisting of GLCM, GLRLM, NGTDM and wavelet transformation features. The surface area to volume ratio was not amongst their feature set, meaning it could be a discriminating feature specific to large nodules. However, our second selected feature, GLCM_Correlation, is common between both models, and could be an important predictor of malignancy. We believe the advantage of our two-feature model, which does not include wavelet transformations, is that it is more readily interpretable and reproducible.

Although the LN-RPV retained good performance in the external test set (accuracy 76%), this data was obtained from public imaging databases which may not closely match the setting in which the algorithm is intended to be used. Therefore additional external testing with large, representative datasets is required before generalisable clinical use. Prospective evaluation in a real-world nodule MDT is the next step to verify its clinical utility.

Aside from the external test set, there are some other limitations to consider. Firstly, the model does not incorporate changes in radiomics features over time, which is an area for future development. Secondly, though we have developed an auto-segmentation pipeline, a truly integrated solution whereby all pre-processing, segmentation and extraction steps are unified into a single program has not yet been developed. Thirdly, a limitation of the clinical decision-support scenario is that imputation of the PET as negative when missing could underestimate Herder score performance. And finally, we note that the LN-RPV was not statistically significantly better than the Herder score using DeLong's test. However, it has been noted by Vickers et al. that the DeLong test is conservative, and that a single test, namely multivariable regression incorporating established variables and the novel predictor, is sufficient to draw conclusions about a new model's utility.^38^ As the LN-RPV retained significance in multivariable testing, and identified cancers within the established Herder category of 10–70%, we believe that meaningful conclusions can be drawn about its utility in the context of established clinical models.

In summary, the LIBRA study has provided a national pipeline for multi-centre lung nodule AI research, which has been used to develop a large nodule classification algorithm for lung cancer diagnosis. Our model appears to perform better than clinical radiologists and the Brock score, and comparably to the Herder score. The modelled decision-support scenarios suggest it could lead to earlier-intervention for malignant nodules in the 10–70% Herder category, which could potentially save lives through early intervention in the future.

## Contributors

B.H.: Study design, data collection, data analysis, manuscript preparation.

M.C., E.A., A.L., B.R.: Radiology reads.

P.R., D.T., N.S., N.N., A.B., S.B., S.K.: Data collection.

K.L-R.: Radiomics pipeline supervision.

L.B., S.J.: Study management and operational oversight.

S.D.: Study design, XNAT pipeline management and data anonymisation.

A.N., S.G., S.K.: Study design, trial-management group.

C.B.: Study statistician.

E.A., A.D., R.W.L.: Study design and oversight, student supervision, manuscript preparation.

The LIBRA study PIs were: S.B., S.V.K, N.N., A.D. and R.W.L.

All authors read and approved the final version of the manuscript. The underlying data were accessed and verified by BH, LB and RL.

## Data sharing statement

The anonymised spreadsheets of radiomics features and clinical outcomes used to generate the LN-RPV model are deposited into the Mendeley database under the accession code https://doi.org/10.17632/rz72hs5dvg.1. The R scripts for model development are provided in notebook format at: https://github.com/dr-benjamin-hunter/LIBRA_Large_Nodules. Access to the source images/data will be considered on request to Dr. Richard Lee.

## Declaration of interests

Dr Navani is supported by a Medical Research Council Clinical Academic Research Partnership (MR/T02481X/1). This work was partly undertaken at the University College London Hospitals/University College London that received a proportion of funding from the Department of Health’s National Institute for Health Research (NIHR) Biomedical Research Centre’s funding scheme (NN). Dr Navani reports honoraria for educational talks or advisory boards from Amgen, Astra Zeneca, Boehringer Ingelheim, Bristol Myers Squibb, Guardant Health, Janssen, Lilly, Merck Sharp & Dohme, Olympus, OncLive, PeerVoice, Pfizer, and Takeda.

Dr Nair receives research grants from the Department of Health’s NIHR Biomedical Research Centre and GRAIL. He has received consulting fees from Aidence BV, Faculty Science Limited and MSD. He has received a travel bursary from Takeda. He participates on advisory boards for Aidence BV and Faculty Science Limited. He has leadership roles within the British Society of Thoracic Imaging, the British Lung Foundation and the NHS England Targeted Lung Health Checks Programme.

Dr Lee is funded by the Royal Marsden NIHR BRC, and Royal Marsden Cancer charity. RL's institution receives compensation for time spent in a secondment role for the lung health check program and as a National Specialty Lead for the National Institute of Health and Care Research. He has received research funding from CRUK, Innovate UK (co-funded by GE Healthcare, Roche and Optellum), SBRI (co-applicant in grants with QURE.AI), RM Partners Cancer Alliance and NIHR (co-applicant in grants with Optellum). He has received honoraria from CRUK.

Professor Devaraj is employed by and has stocks in Brainomix. He receives consulting fees from Roche and Boehringer Ingelheim.

The other authors report no potential conflict of interest.
