# Supplementary material for: A radiomics-based decision support tool improves lung cancer diagnosis in combination with the Herder score in large lung nodules
Source: eBioMedicine. 2022 Nov 10;86:104344. doi: 10.1016/j.ebiom.2022.104344 (PMC9664396; doi:10.1016/j.ebiom.2022.104344)
Supplement: Supplementary Figures S1–S4 and Tables S1 and S2 [file mmc1.docx]

| **Characteristic** | **Value** |
| --- | --- |
| **Nodule size** (mean, SD) | 20·63 (4·97) |
| **Nodule Density** (n, %)  Solid  Sub-solid  GGO | 129 (85·4)  21 (13·9)  1 (0·7) |
| **Spiculation** (n, %)  Yes  No | 110 (72·8)  41 (27·2) |
| **Malignancy** (n, %)  No  Yes | 23 (15·2)  128 (84·8) |

**Supplementary Table 1: Patient demographic features for the external test set** (presented at the nodule level). The external test set (n=151) was comprised of data from the LIDC, LUNGx and NSCLC radiogenomics studies. Abbreviations: SD: Standard Deviation, GGO: ground-glass opacity.

| **Metric** | **R1** | **R2** | **R3** |
| --- | --- | --- | --- |
| Accuracy | 65% (95% CI 59 – 71%) | 65% (95% CI 58 – 71%) | 71% (95% CI 65 – 77%) |
| Sensitivity | 0·50 | 0·56 | 0·76 |
| Specificity | 0·91 | 0·80 | 0·63 |
| PPV | 0·91 | 0·82 | 0·77 |
| NPV | 0·52 | 0·52 | 0·61 |
| F1 | 0·64 | 0·66 | 0·77 |

**Supplementary Table 2: Malignancy prediction performance metrics for the three radiologists.** Predictions were obtained using a cutoff of 4 – Probably malignant.

**
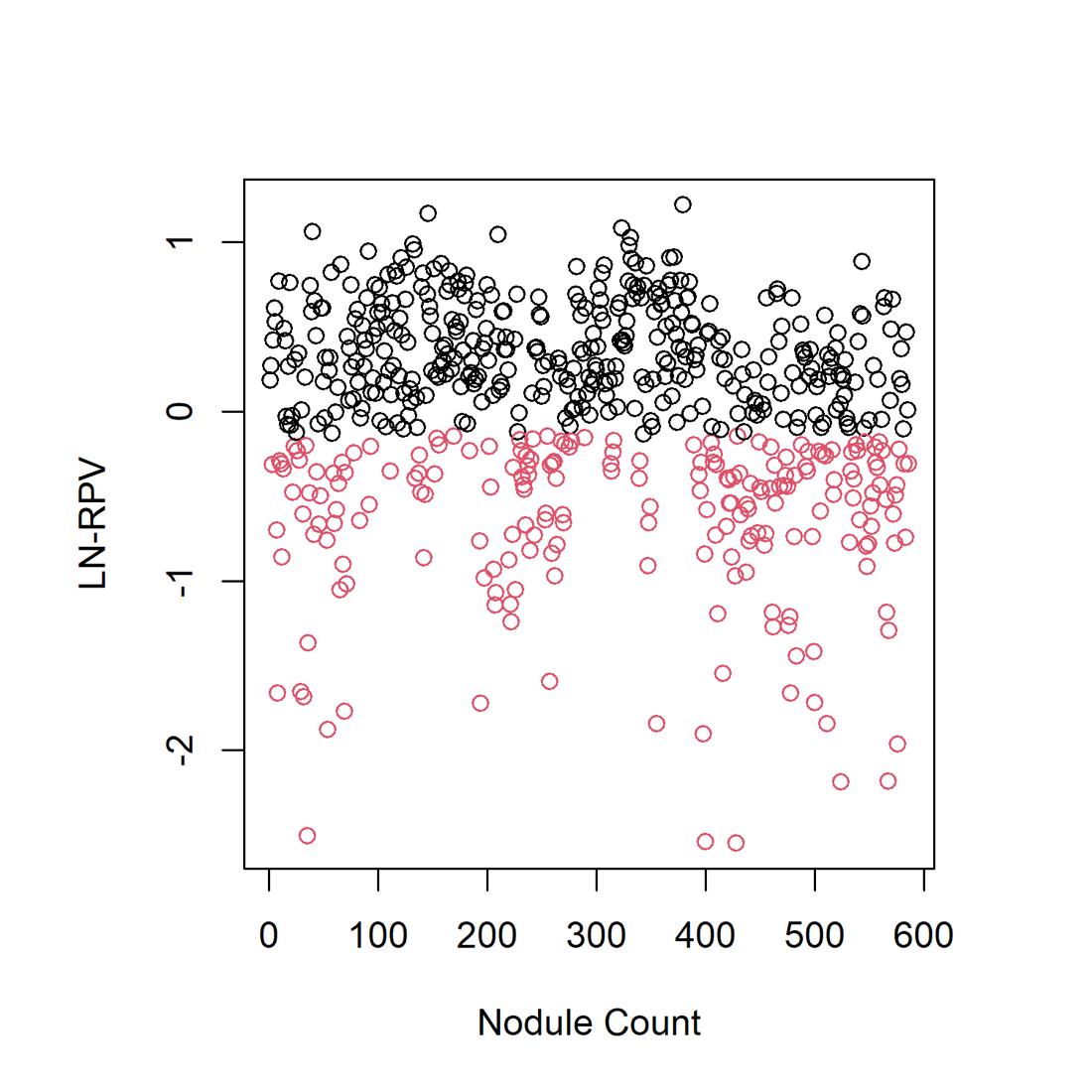
**

**Supplementary Figure 1:** K-means clustering thresholds applied to the training-set LN-RPV (large-nodule radiomics predictive vector) to create low (red) and high (black) risk groups.


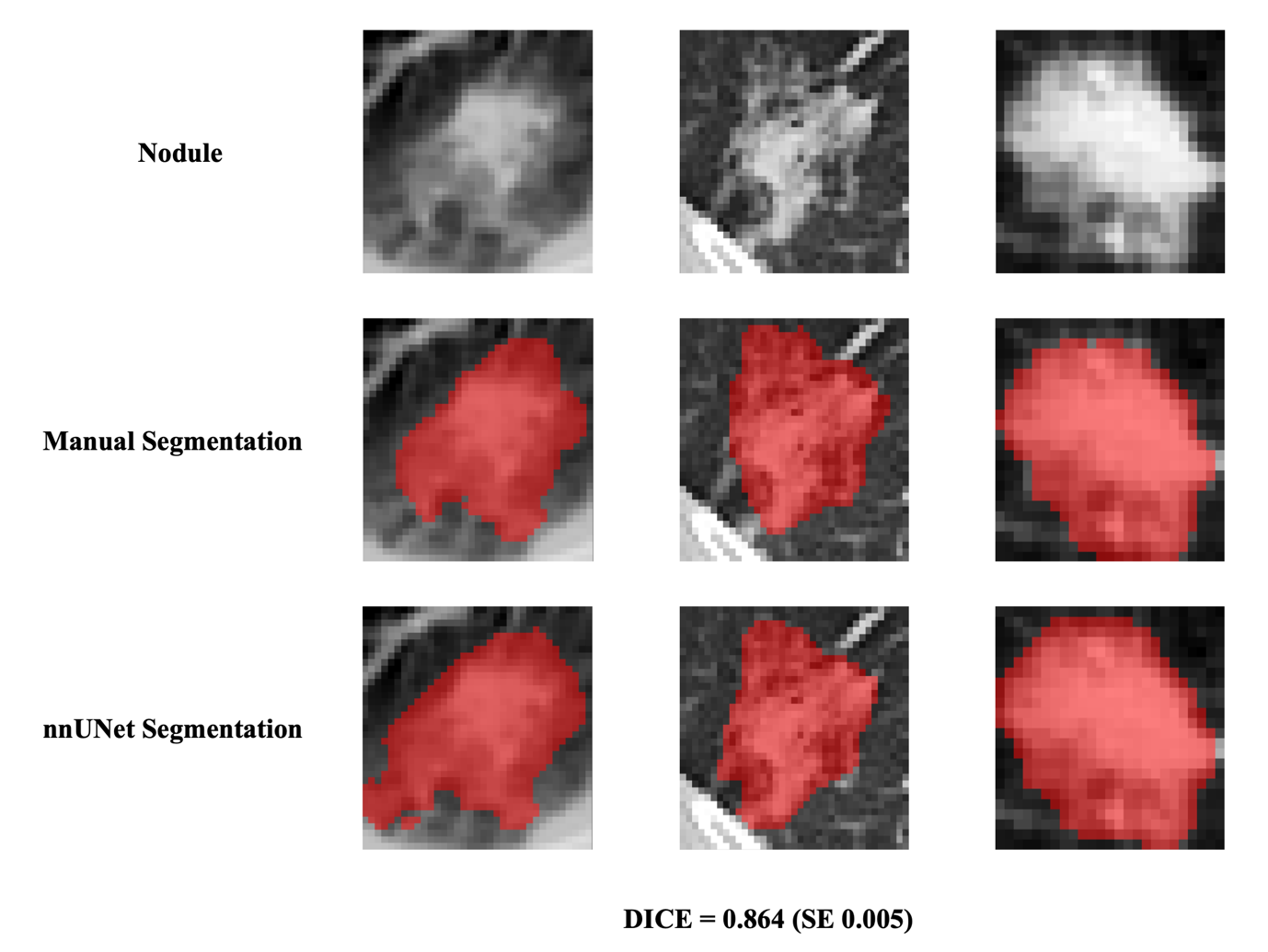


**Supplementary Figure 2: Test set manual and nnUNet segmentation masks** (n=252). The auto-segmentation model DICE score was 0·86 in the test set. Abbreviations: SE: standard error.


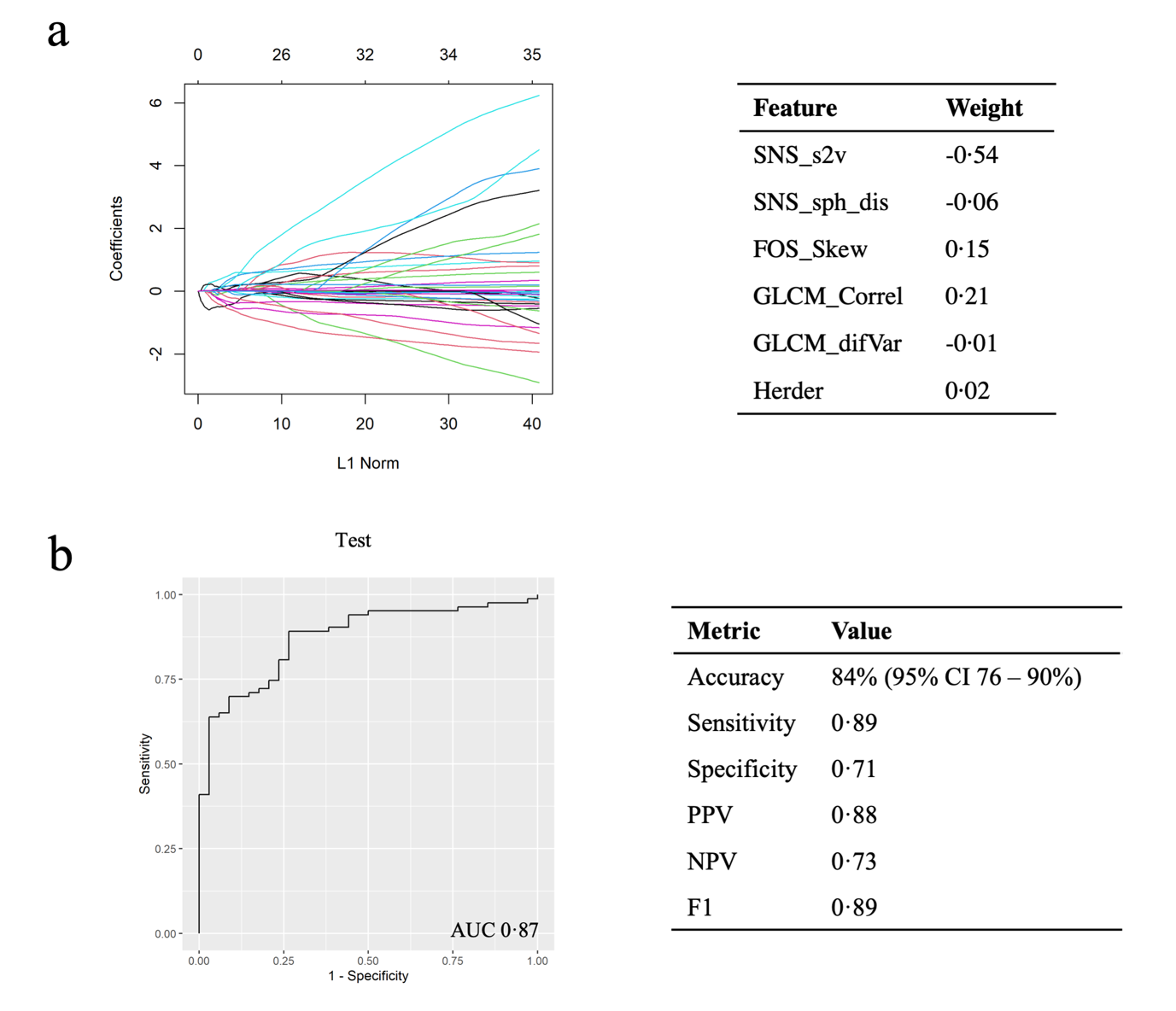


**Supplementary Figure 3: An ‘early’ fusion model providing radiomics features and the Herder score at the LASSO step.** a) The LASSO logistic regression model selected 6 features with non-zero coefficients, including 5 radiomics features and the Herder score (n=586). b) The fusion model was tested on baseline solid nodules in the test set (n=117), and achieved an AUC of 0·87 (0·80-0·93). This AUC was equal to that of the LN-RPV, and was not statistically significantly better than the Herder score (DeLong’s p = 0·08).


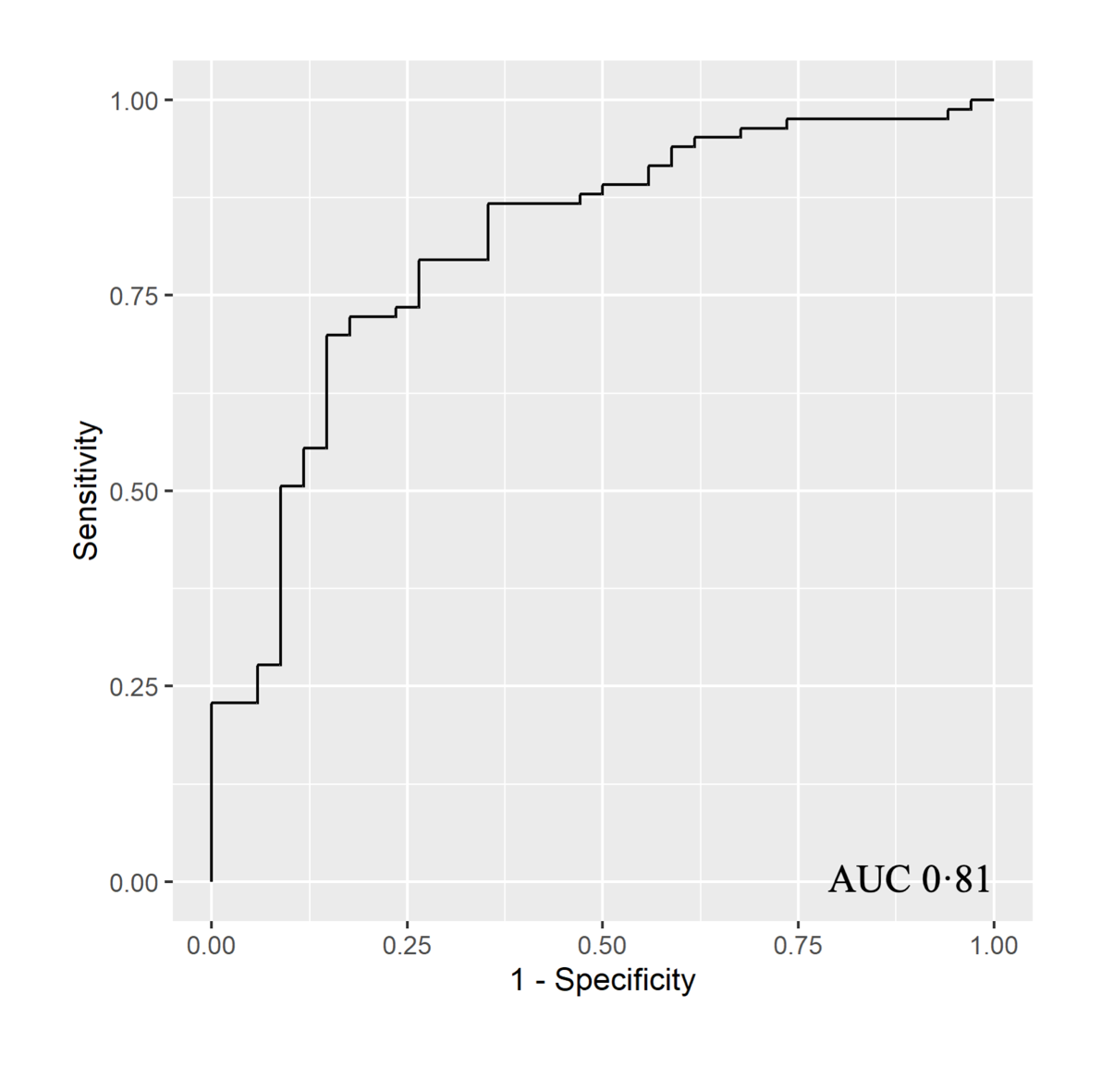


**Supplementary Figure 4: ROC curve for an XGBoost fusion model including the Herder score and the LN-RPV** (solid nodules in the test set, n = 117). The XGBoost fusion model was not statistically significantly better than the Herder score alone (DeLong’s p = 0·77).
