# Supplementary material for: A radiomics-based decision support tool improves lung cancer diagnosis in combination with the Herder score in large lung nodules
Source: eBioMedicine. 2022 Nov 10;86:104344. doi: 10.1016/j.ebiom.2022.104344 (PMC9664396; doi:10.1016/j.ebiom.2022.104344)
Supplement: Supplementary File S1 [file mmc2.docx]

**Supplementary Table 1: Patient demographic features for the external test set** (presented at the nodule level). The external test set (n=151) was comprised of data from the LIDC, LUNGx and NSCLC radiogenomics studies. Abbreviations: SD: Standard Deviation, GGO: ground-glass opacity.

**Supplementary Table 2: Malignancy prediction performance metrics for the three radiologists.** Predictions were obtained using a cutoff of 4 – Probably malignant.

**Supplementary Figure 1:** K-means clustering thresholds applied to the training-set LN-RPV (large-nodule radiomics predictive vector) to create low (red) and high (black) risk groups.

**Supplementary Figure 2: Test set manual and nnUNet segmentation masks** (n=252). The auto-segmentation model DICE score was 0.86 in the test set. Abbreviations: SE: standard error.

**Supplementary Figure 3: An ‘early’ fusion model providing radiomics features and the Herder score at the LASSO step.**

**Supplementary Figure 4: ROC curve for an XGBoost fusion model including the Herder score and the LN-RPV.**
